# Cytotoxicity of Particulate Matter PM_10_ Samples from Ouagadougou, Burkina Faso

**DOI:** 10.1155/2022/1786810

**Published:** 2022-10-21

**Authors:** Joelle Nicole Guissou, Isabelle Baudrimont, Abdoul Karim Ouattara, Jacques Simpore, Jean Sakande

**Affiliations:** ^1^Laboratory of Environment and Health Toxicology (LATES), University Joseph Ki-Zerbo, 03 BP 7021, Ouagadougou 03, Burkina Faso; ^2^Cardiothoracic Research Center of Bordeaux (CRCTB) INSERM-U1045, Ptib-Hospital Xavier Aozanrn, Avenue Du Haut Leveque, Pessac 33604, France; ^3^Center for Biomolecular Research Pietro Annigoni (CERBA), 01 BP 364, Ouagadougou 01, Burkina Faso; ^4^Health Department, Laboratory of Biochemistry, University Joseph Ki-Zerbo, 03 BP 7021, Ouagadougou 03, Burkina Faso

## Abstract

Particulate matter (PM) is one of the main air pollutants with 257,000 deaths per year in Africa. Studying their toxic mechanisms of action could provide a better understanding of their effects on the population health. The objective of this study was to describe the PM_10_ toxic mechanism of action collected in 3 districts of Ouagadougou. Once per month and per site between November 2015 and February 2016, PM_10_ was sampled for 24 hours using the MiniVol TAS (AirMetrics, Eugene, USA). The collected filters were then stored in Petri dishes at room temperature for in vitro toxicological studies using human pulmonary artery endothelial cells (HPAEC) at the Bordeaux INSERM-U1045 Cardio-thoracic Research Center. The three study districts were classified based on PM_10_ level (high, intermediate, and low, respectively, for districts 2, 3, and 4). PM_10_ induced a concentration-dependent decrease in cell viability. A significant decrease in cell viability was observed at 1 *µ*g/cm^2^, 10 *µ*g/cm^2^, and 25 *µ*g/cm^2^ for, respectively, districts 2, 3, and 4. A significant increase in the production of reactive oxygen species (ROS) was observed at 10 *µ*g/cm^2^ for district 2 versus 5 *µ*g/cm^2^ and 1 *µ*g/cm^2^ for districts 3 and 4, respectively. Finally, a significant production of IL-6 was recorded from 5 *µ*g/cm^2^ for district 4 versus 10 *µ*g/cm^2^ for districts 2 and 3. Consequently, Ouagadougou is subjected to PM_10_ pollution, which can induce a significant production of ROS and IL-6 to cause adverse effects on the health of the population.

## 1. Introduction

Air pollution is one of the major environmental risk factors for the population health [1]. According to the WHO, a reduction in air pollution levels results in a significant decrease in morbidity from cardiovascular and respiratory diseases, including asthma [2]. The estimated number of deaths due to air pollution was 4.2 million worldwide in 2016 [2]. Most epidemiological studies in air pollution have been conducted in developed countries, and particulate matter (PM) is the main cause [3, 4].

The level of PM is an indicator of air pollution, and their size during inhalation exposure seems to determine the affected target organs as well as their impact on health [5]. Indeed, PM_2.5_, fine particles with an aerodynamic diameter less than 2.5 *µ*m, penetrate deeply into the lungs to the terminal bronchioles and alveoli, whereas PM_10_, with a diameter of 10 *µ*m, are deposited mainly in the upper respiratory tract [5, 6]. Autopsies performed on London smog air pollutant victims in 1952 revealed a high occurrence of chronic obstructive pulmonary disease (COPD) [7]. Epidemiological studies have shown the relationship between exposure to PM air pollution and pulmonary pathology (asthma exacerbation, pulmonary cancer, and COPD) [8, 9].

Regarding the significant consequences of air pollution, the WHO organized the first international conference on air pollution in 2018 [10]. However, Africa has few air pollution studies [11]; although, the United Nations International Children's Emergency Fund (UNICEF) recorded 258,000 deaths due to air pollution in 2017 in Africa [12]. Sub-Saharan African populations are the most affected by the air quality degradation effects [13]. Unfortunately, interest in air quality is of lesser importance due to the lack of political will and the absence of adequate tools for air quality monitoring [11]. Ouagadougou, the capital of Burkina Faso, is not excluded from this situation.

Located in the heart of the Sahel, Ouagadougou has, in addition to the factors mentioned above, a growing fleet of very old and poorly maintained vehicles in a context of increasing human activity [14]. Studies on air pollution are rare, incomplete, or old. They are mainly physicochemical studies on pollutants for environmental pollution [14–18]. Studies on PM_10_ air pollution considered to be of concern for the health of populations and understanding their toxicological mechanisms of action at the cellular level are needed to guide decision-making about air pollution. In the current study, we report a possible toxicological mechanism of PM_10_ action collected in Ouagadougou on pulmonary vascular target cells.

## 2. Materials and Methods

### 2.1. Cell and Culture Conditions

PromoCell® human pulmonary artery endothelial cells (HPAECs) were used for all toxicology studies. The cells were grown in monolayers in 25 cm^2^ culture flasks (Falcon®) at a density of 20,000 cells/cm^2^ in Complete Red Medium (CRM).

CRM consisted of Endothelial Cell Growth Medium (ECGM), with phenol red, without antibiotics or fungicides, supplemented with Supplement Mix (PromoCell®). The Supplement Mix contains 2% fetal bovine serum (FBS), 0.4% endothelial cell growth supplement (ECGS), 0.1 ng/mL epidermal growth factor (EGF), 1 ng/mL basic fibroblast growth factor (BFGF), 90 *µ*g/mL heparin, and 1 *µ*g/mL hydrocortisone.

Cells were incubated at 37°C in a controlled humid atmosphere of 5% CO_2_ at pH = 7.4. The culture medium was changed every other day. When the cells reached 80% confluence, they were subcultured. The cells were used between passages 2 and 8. Experiments with the cells were performed in a sterile environment under a laminar flow hood in a P2 laboratory.

### 2.2. Atmospheric Samples

PM_10_ can directly penetrate the respiratory tract and alveoli to cause adverse health effects in the exposed population. The PM_10_ level was evaluated in three districts of Ouagadougou, and the districts were then classified based on the PM_10_ level (high PM_10_ level, intermediate PM_10_ level, and low PM_10_ level). Particulate samples from each category (district 2 for high PM_10_, district 3 for intermediate PM_10_, and district 4 for low PM_10_) were used for the toxicological studies. The MiniVol TAS (AirMetrics, Eugene, USA), a portable air sampling device, was used to sample PM_10_ from ambient air with a Teflon filter. The filters were weighed before and after sample collection. The device was placed on a stand at the height of a 1.70 m human airway. An authorization (ref: 2016/Labio/XI-02/0135) was requested from the competent structures of the different districts for the PM_10_ sampling. Four sampling campaigns (November 2016, December 2016, January 2017, and February 2017) were scheduled at each site within the different districts.

The mixture from the different campaigns per district was used as the sample from each district. The filters were stored in Petri dishes at room temperature for *in vitro* toxicology studies. The experimental (toxicological) study was performed at the Cardio-thoracic Research Center of Bordeaux (CRCTB) INSERM-U1045.

### 2.3. Extraction of Particles from Filters

The protocol used in the present study [19] has been adapted by the CRCTB (INSERM U 1045, University of Bordeaux) and the laboratory UMR-CNRS 8251 (University Paris Diderot) [20]. The filters without their periphery were cut into four pieces. Each piece was then placed in Eppendorf tubes supplemented with 500 *µ*l of Complete White Medium (CWM), antibiotics (1% penicillin/streptomycin), and antifungals (1% amphotericin B).

The Eppendorf was then alternately vortexed and sonicated (Vibracell® 75186) using an induction probe. Then, the filter was removed and placed in a second Eppendorf tube containing 700 *µ*l of CWM supplemented with antibiotics and antifungals. The same process was also applied to the second Eppendorf. Once all filter pieces were processed, the contents of the two Eppendorf tubes were combined to have a total volume of 1200 *µ*l. The samples were then stored at −20°C for later use.

### 2.4. Cytotoxicity Assessment (WST-1 Test)

The cytotoxicity of PM_10_ was evaluated by the water-soluble tetrazolium test (WST-1). This colorimetric test is based on the cleavage of the colorless tetrazolium salts WST-1 (4-[3-(4-iodophenyl)-2-(4-nitrophenyl)-2H-5 tetrazolio]-1, 3-benzene disulfonate) into a formazan derivative, soluble in yellow color by mitochondrial succinate dehydrogenases of living cells. HPAEC cells were first seeded in ninety-six-well plates at a density of 20,000 cells/cm^2^ and cultured for 24 h at 37°C with 5% CO_2_. They were subsequently exposed to different concentrations of PM_10_ (1 *µ*g/cm^2^, 5 *µ*g/cm^2^, 10 *µ*g/cm^2^, 25 *µ*g/cm^2^, and 50 *µ*g/cm^2^). The experiment was repeated three times with four wells per condition. After 24 hours of exposure, the cells are rinsed with CWM supplemented with antibiotics and antifungals and incubated for 3 hours at 37°C with 5% CO_2_ with CWM supplemented with antifungals and antibiotics containing 10% of WST-1 [21].

Absorbance was measured directly by spectrophotometry at 450 nm corrected to 630 nm, using a SPECTROstarNano2.10 plate reader and MARS Data Analysis Software (BMG LabTech®). The toxicity assessment determined the PM_10_ concentrations to be used for the mechanistic studies. It was agreed not to exceed 10 *µ*g/cm^2^, a concentration resulting in less than 30% cell death. This choice was also made for this concentration to remain as realistic as possible regarding the concentrations to which humans could be exposed.

### 2.5. Assessment of the Production of Reactive Oxygen Species

Oxidative stress levels follow different steps over time, from antioxidant defense to cytotoxicity through inflammation [22]. Therefore, the ROS production was assessed after 4 h exposure of cells to PM_10_ according to the method adapted from Deweirdt et al. [21].

The different reactive oxygen species (ROS) were quantified by spectrofluorimetric method using the CM-H_2_DCF-DA probe (5-(and-6)-chloromethyl-2′,7′-dichlorodihydrofluorescein diacetate, acetyl ester), Fisher Scientific®). Nonfluorescent H_2_DCFCM-H_2_DCF-DA CM-H2DCF-DA in the acetylated and reduced state is cleaved at the 2′ and 7′ acetylated groups by an esterase to yield nonfluorescent H_2_DCF. The molecule is then oxidized by ROS to a fluorescent derivative, DCF, which can easily be quantified by fluorimetry. Thus, the fluorescence intensity is directly proportional to the amount of ROS formed in the cell [23].

The probe and cells were protected from light. Cells were seeded into twenty-four-well plates at a density of 20,000 cells/cm^2^ and cultured for 24 hours at 37°C with 5% CO_2_. Prior to exposure, cells were rinsed and incubated for 20 minutes at 37°C in 5% CO_2_ with the probe (20 *µ*M) solubilized in serum-free blank medium. Once excess probe not incorporated by the cells had been removed, the cell layer was rinsed with CWM supplemented with antibiotics and antifungal at 1% and the cells were exposed to different concentrations of PM_10_ such as 1 *µ*g/cm^2^, 5 *µ*g/cm^2^, and 10 *µ*g/cm^2^ from Ouagadougou districts 2, 3, and 4. The experiment was replicated three times (*n* = 3) with three wells per condition. The production of ROS was measured in HPAEC by spectrofluorimetry (probe CM-H_2_DCFDA) after 4 hours of exposure.

Fluorescence intensity was determined using a plate reader (FLUOstar Omega 2.10 and MARS Data Analysis Software 2.30 R3 (BMG LabTech®) at an excitation wavelength of 485 nm and an emission of 520 nm. The results were expressed as a percentage increase in ROS compared to control cells.

### 2.6. Interleukin-6 (IL-6) Assay

Studies have shown that PM_10_ induces the production of cytokines such as TNF-*α*, IL-1, IL-6, IL-8, or IL-10. However, IL-6 is a proinflammatory cytokine that intervenes during the acute phase of inflammation. Previous studies also demonstrated a correlation between IL-6 production and PM_10_ exposure [24–26]. Based on these previous studies, we focused here on the assessment of IL-6 secretion.

Cells were seeded in twelve-well plates at a density of 20,000 cells/cm^2^ and cultured for 24 hours at 37°C with 5% CO_2_. Before exposure, the cell mat was rinsed. Then the cells were contacted with different PM_10_ concentrations (1.5 and 10 *μ*g/cm^2^). The experiment was repeated three times (*n* = 3) with three wells per condition. After 24 hours of exposure, the supernatants were removed, centrifuged (10 minutes, at 15,000 g and at 4°C), and stored at −20°C until assay.

The assay of IL-6 cytokines in culture supernatants was performed by the enzyme-linked immunosorbent assay (ELISA) technique using DuoSet kits (R&D Systems®), a SPECTROstarNano2.10 plate reader, and MARS 2.41 Data Analysis Software (BMG LabTech®). To relate the IL-6 cytokine level (ELISA) to the protein levels, a Lowry test was performed in the culture supernatants for each condition, using the Biorad® DC Protein assay reagent package kit. Absorbance was measured using a SPECTROstarNano2.10 plate reader 2.41 data analysis software (LabTech®) at 750 nm. For each test, the results are expressed as means ± the standard error of the mean (SEM). Each experiment was performed independently three times (*n* indicates the number of experiments, *n* = 3). IL-6 was assayed in the culture supernatant of HPAEC cells after 24 hours of intoxication at different concentrations of PM_10_ (1.5 and 10 *µ*g/cm^2^) in districts 2, 3, and 4.

### 2.7. Statistical Analyses

Differences between the means of HPAEC control and PM_10_-exposed cells were evaluated using different tests: an ANOVA parametric test followed by a multiple comparison test with Dunnett's correction. Statistical analyses and graphs were performed with GraphPad Prism 5.0 software. Differences were considered significant when *p* < 0.05(^*∗*^), *p* < 0.01(^*∗∗*^), and *p* < 0.001(^*∗∗∗*^).

## 3. Results

### 3.1. The Effect of PM_10_ on Cell Viability is District-Specific and Concentration-Dependent

**Figure 1:**
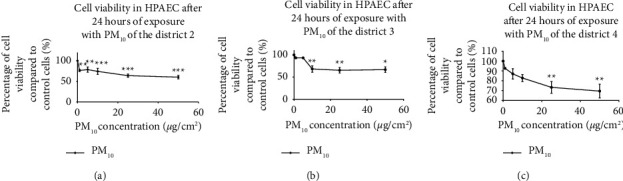
Effects of PM_10_ from districts 2 (a), 3 (b), and 4 (c) of Ouagadougou (Burkina Faso) on the HPAEC cell viability after 24 hours of exposure. The HPAEC cells were seeded at 20,000 cells/cm^2^ and then intoxicated or not for 24 hours at different concentrations of PM_10_ (1.5, 10, 25, and 50 *µ*g/cm^2^). Cell viability was measured by the WST-1 assay. Percent cell viability was calculated relative to control cells (100%). Data are expressed as means ± the standard error of the mean (SEM) of three independent experiments *n* = 3 (three wells/concentration). The statistical test used is a one-way ANOVA followed by a multiple comparison with Dunnett's correction ^*∗*^*p* < 0.05, ^*∗∗*^*p* < 0.01, and ^*∗∗∗*^*p* < 0.001: significance level compared to control cells.

The nature and chemical composition of PM_10_ are important factors in their health effects. The current study results show that after 24 hours of exposing HPAEC cells to different PM_10_ concentrations, there is a significant concentration-dependent decrease in cell viability from 1 *µ*g/cm^2^ for district 2 (Figure 1(a); 76.5% ± 2.5% cell viability), 10 *µ*g/cm^2^ for district 3 (Figure 1(b); 68.4% ± 7.2% cell viability), and 25 *µ*g/cm^2^ for district 4 (Figure 1(c); 73.3% ± 6% cell viability). At the highest concentration (50 *μ*g/cm^2^), a viability of 60.3% ± 4.1%, 66.9% ± 6.4%, and 69.5% ± 7% was noted, respectively, for districts 2, 3, and 4. A significant difference was observed at the concentration of 1 *µ*g/cm^2^ in HPAEC cells for districts 2 and 3 (76.5% ± 2.5% versus 93.4% ± 1.2% and for districts 2 and 4 (76.5% ± 2.5% versus 92.6% ± 0.39%). Overall, the results demonstrate that increasing the concentration decreases cell viability, and the threshold concentration to obtain a significant decrease in cell viability varies from one district to another.

### 3.2. PM_10_-Induced ROS Production is District-Specific and Concentration-Dependent

**Figure 2:**
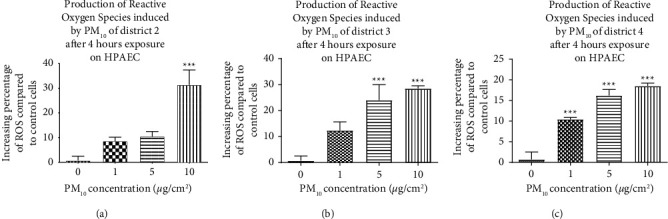
Production of ROS in HPAEC cells after 4 hours of exposure to PM_10_ of districts 2 (a), 3 (b), and 4 (c) of Ouagadougou (Burkina Faso). The HPAEC cells were seeded for 24 hours and then treated with different concentrations of PM_10_ (1.5, 10, 25, and 50 *µ*g/cm^2^) for 4 hours. Intracellular ROS production was evaluated by the CM-H2DCFDA probe and measured by spectrofluorimetric method. The percentage increase in ROS is calculated relative to control cells. Data are expressed as means ±SEM of three independent experiments *n* = 3 (three wells per concentration). The statistical test used is a one-way ANOVA followed by a multiple comparison with Dunnett's correction, ^*∗∗∗*^*p* < 0.001: significance level compared to control cells.

PM_10_-induced reactive oxygen species (ROS) production is concentration-dependent. The results show a concentration-dependent increase in ROS production in HPAEC cells compared to control cells (Figure 2).

This increase is significant at the highest concentration (10 *µ*g/cm^2^) for district 2 (Figure 2(a); 31.4% ± 6.1% increase in ERO production), whereas for districts 3 and 4 (Figure 2(b)) and 4 (Figure 2(c)), a significant increase is observed from 5 *μ*g/cm^2^ (24.1% ± 6% increase in ERO production) and from 1 *μ*g/cm^2^ (10.4% ± 0.4%), respectively. A comparison was made at the highest concentration (10 *µ*g/cm^2^) for the three districts. No significant difference was detected in ROS production in HPAEC cells after 4-hour exposure.

### 3.3. PM_10_-Induced IL-6 Secretion is District-specific and Concentration-Dependent

**Figure 3:**
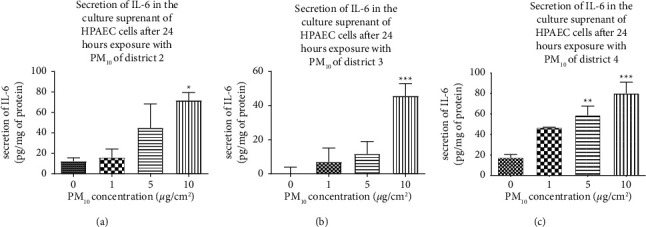
Secretion of the proinflammatory cytokine IL-6 in HPAEC cells after 24 hours of exposure to PM_10_ from districts 2 (a), 3 (b), and 4 (c) of Ouagadougou (Burkina Faso). HPAEC cells were seeded for 24 hours and then treated with different concentrations of PM10 (1, 5, and 10 *µ*g/cm2) for 24 hours. The IL-6 concentration (pg/mL) was determined by using the ELISA technique and measured by spectrophotometry. This concentration was then related to total protein levels for each well (pg/mg). Data are expressed as the mean ± SEM of three independent experiments *n* = 3 (three wells/concentration). The test used is a one-way ANOVA followed by a multiple comparison with Dunnett's correction ^*∗*^*p* < 0.05: Significance level compared to control cells.

The results reveal a concentration-dependent increase in IL-6 after a 24-hour exposure to PM_10_ in all three districts of Ouagadougou. This increase is significant compared to control cells at the highest concentration (10 *μ*g/cm^2^) for districts 2 (Figure 3(a)) and 3 (Figure 3(b)). At this concentration, the mean secretion of IL-6 was 317.2 pg/mg ± 38.2 pg/mg and 272.7 pg/mg ± 32.27 pg/mg, respectively for districts 2 and 3, that is, an increase of 71.8% ± 7.8% and 45.7% ± 7.2%, respectively, compared to control cells. The increase was significant for district 4 (Figure 3(c)) from 5 *µ*g/cm^2^ with a mean IL-6 secretion of 222.5 pg/mg ± 43.14 pg/mg, an increase of 58.8% ± 9% relative to control cells. A comparison was performed at a concentration of 10 *µ*g/cm^2^ for the three districts. A significant difference was observed between districts 3 and 4.

## 4. Discussion

The PM_10_ collected in each of the three districts in the current study led to a concentration-dependent decrease in cell viability. Our results are consistent with those of Dieme et al., who had shown the ability of particles collected in Dakar (Senegal) to alter mitochondrial metabolism [27]. Many studies have also shown a concentration-dependent decrease in cell viability following exposure of epithelial cells to PM_10_ [28]. The nature and chemical composition of PM_10_ certainly determine the threshold amount toxic for cells.

Our results show that after 24 hours of exposure to PM_10_, a significant (concentration-dependent) decrease in cell viability was observed from 1 *µ*g/cm^2^ for district 2. Cachon et al. in a study conducted on particles from Cotonou (Benin) had shown a significant decrease in cell viability from 3 *µ*g/cm^2^ [29]. The similarity of these results could be explained by the fact that Cotonou and Ouagadougou are two cities with comparable profiles (transportation, human activities, and city expansion). In our study, the significant decrease in cell viability was observed from 10 *µ*g/cm^2^ for district 3 and from 25 *µ*g/cm^2^ for district 4. Indeed, district 2 is a district with a high level of PM_10_, while district 3 has a lower level of PM_10_ than the first one. Only a difference in PM_10_ chemical composition could explain the significant difference observed from 25 *µ*g/cm^2^. When comparing the cell viability of the three districts, for an exposure to a PM_10_ concentration of 10 *µ*g/cm^2^, significant differences were observed between districts 2 and 3 and between districts 2 and 4. Toxicity terms are noted between the different districts depending on the PM_10_ profiles.

Redox homeostasis is essential for cell function and viability [30]. Exposure to extrinsic stress factors such as PM can disrupt this balance. To assess whether the PM_10_ samples collected in different districts of Ouagadougou could disrupt this balance, the production of ROS was studied. The results show that after 4 hours of exposure, there is a concentration-dependent increase in ROS production in HPAEC cells compared to control cells. The literature reports similar results found in neighboring countries of Burkina and throughout the world [29, 31, 32]. A previous study [20] conducted in Ouagadougou on a single sampling point within the framework of the French National Research Agency MEGATOX project in 2010 also showed a concentration-dependent increase in ROS production in HPAEC cells.

At the highest concentration (10 *µ*g/cm^2^), the increase in ROS production was approximately 16% in the MEGATOX project sample [20] compared to 31.4% ± 6% for district 2, 28.5% ± 1.1% for district 3, and 18.5% ± 0.7% for district 4. The values found in the present study are much higher than the previous studies of MEGATOX project. These results could be explained by an increase in PM pollution in recent years and changes in the chemical composition of PM_10_. Indeed, between 2010 and 2017, the city of Ouagadougou experienced a significant development of anthropogenic activities that constitute a major pollution factor [33]. The comparison of ROS production induced by PM_10_ between different districts revealed a significant increase in ROS production at the concentration of 10 *µ*g/cm^2^ for district 2, whereas for districts 3 and 4, a significant increase was observed from 5 *µ*g/cm^2^. ROS production can be due to several mechanisms [34]. The study focused on the assessment of ROS production using the CM-H_2_DCFH-DA probe, which allows the evaluation of intracellular changes in H_2_O_2_. Other methods for evaluating oxidative stress such as the glutathione assay or the lipid peroxidation assay could explain this difference in results. Indeed, the difference in ROS production induced by PM_10_ collected in districts 3 and 4 could be due to these two mechanisms. A difference in the chemical composition of PM_10_ could also explain our results [1].

It has been shown in the literature that oxidative stress could trigger a proinflammatory response [35]. To assess these hypotheses, our cellular model was treated with PM_10_ from the three districts of Ouagadougou, and the secretion of proinflammatory cytokines such as IL-6 was evaluated. The results show a concentration-dependent increase in IL-6 after 24 hours of PM_10_ treatment. Ndong Ba et al. in a study carried out on BEAS-2B cells treated with PM from Dakar (Senegal) also found a significant dose-dependent increase in IL-6 production of about 750 pg/ml from 3 *µ*g/cm^2^ for urban sites and approximately 250 pg/ml from 3 *µ*g/cm^2^ for rural sites [31]. The results of the present study were significant for districts 2 and 3, at a concentration of 10 *µ*g/cm^2^ with a rate of 317.2 pg/mg ± 38.2 pg/mg and 272.7 pg/mg ±  32.27 pg/mg, respectively. For district 4, the results were significant from 5 *µ*g/cm^2^ with a value of 222.5 pg/mg ± 43.14 pg/mg. These differences could be due to the variable chemical composition between the PM_10_ from Ouagadougou and those from Dakar. A significant difference was found between districts 3 and 4, after comparing the inflammatory response induced by PM_10_ collected in these districts at the concentration of 10 *μ*g/cm^2^. This is in line with the classification of districts by PM_10_ level with district 3 as PM_10_ low level area and district 4 as PM_10_ intermediate level area. Overall, the results of the present study suggest an association between PM_10_ exposure and the occurrence of pulmonary disease. The toxicological study also supports this hypothesis through the correlation between PM_10_ exposure and ROS production and the secretion of proinflammatory mediators (IL-6). Despite limitations such as the short collection period, the selection of patients based on voluntary participation, and the failure to evaluate some toxicological mechanisms of action, the present original study is of high relevance in a country such as Burkina Faso to document the toxicological profile of atmospheric pollution with PM_10_.

## 5. Conclusions

The results of the present study revealed that PM_10_ induced dose-dependent cytotoxicity to a greater or lesser extent by sampling district. Oxidative stress is a key source of toxic substances found in the endothelial cells of human pulmonary arteries that can trigger a proinflammatory response. PM_10_, unlike larger particles that are filtered by the nasal and bronchial cilia, directly penetrate the upper respiratory tract and alveoli, causing inflammation and irritating the bronchi, thus affecting lung function, and exacerbating symptoms of respiratory diseases and other pathologies according to the intensity or duration of the inflammation.

The results of this study lead us to question the profile of atmospheric pollution by particulate pollutants in Ouagadougou (Burkina Faso), especially the other types of PM found there and their chemical composition. In addition, it would be interesting to conduct a study to better define the clinical effects of PM. Finally, to confirm our assumption, it would be necessary, through further studies, to better characterize, in the endothelial cells of human pulmonary arteries, the mechanisms responsible for the toxicity of PM and to assess whether oxidative stress could be a source of calcium signaling alterations or an apoptotic process.

## Data Availability

The datasets used and/or analyzed during the current study are available from the corresponding author on reasonable request.
